# Deep Learning-Based Analysis of Face Images as a Screening Tool for Genetic Syndromes

**DOI:** 10.3390/s21196595

**Published:** 2021-10-02

**Authors:** Maciej Geremek, Krzysztof Szklanny

**Affiliations:** 1 Department of Medical Genetics, Institute of Mother and Child, 01-211 Warsaw, Poland; maciej.geremek@imid.med.pl; 2 Multimedia Department, Polish-Japanese Academy of Information Technology, 02-008 Warsaw, Poland

**Keywords:** dysmorphic features detection, face recognition, genetic disease, DNN, classifier

## Abstract

Approximately 4% of the world’s population suffers from rare diseases. A vast majority of these disorders have a genetic background. The number of genes that have been linked to human diseases is constantly growing, but there are still genetic syndromes that remain to be discovered. The diagnostic yield of genetic testing is continuously developing, and the need for testing is becoming more significant. Due to limited resources, including trained clinical geneticists, patients referred to clinical genetics units must be accurately selected. Around 30–40% of genetic disorders are associated with specific facial characteristics called dysmorphic features. As part of our research, we analyzed the performance of classifiers based on deep learning face recognition models in detecting dysmorphic features. We tested two classification problems: a multiclass problem (15 genetic disorders vs. controls) and a two-class problem (disease vs. controls). In the multiclass task, the best result reached an accuracy level of 84%. The best accuracy result in the two-class problem reached 96%. More importantly, the binary classifier detected disease features in patients with diseases that were not previously present in the training dataset. The classifier was able to generalize differences between patients and controls, and to detect abnormalities without information about the specific disorder. This indicates that a screening tool based on deep learning and facial recognition could not only detect known diseases, but also detect patients with diseases that were not previously known. In the future, this tool could help in screening patients before they are referred to the genetic unit.

## 1. Introduction

Rare diseases occur with a population frequency of less than 1:2000 [1], and since they encompass more than 8000 forms, more than 30 million patients suffer from rare diseases in Europe alone. More than 72% of these disorders have a genetic background. The human genome encodes more than 22,000 genes, and almost 7500 of them have been linked to human diseases [2]. The diagnostic yield of genetic testing has substantially evolved from targeted single gene sequencing to next generation sequencing (NGS) that enables the analysis of thousands of genes in one experiment. However, the analytical abilities are still limited due to extensive genetic variability and large amounts of data generated by these technologies. A correct interpretation of the sequencing data still has to be based on the clinical evaluation of the patient. 

Genetic diseases can affect any system or organ in the human body. Around 30–40% of these disorders are associated with specific facial characteristics called dysmorphic features [3]. In many cases, the dysmorphic features result from abnormal embryological development, i.e., in craniosynostosis, gene mutations cause a premature closure of cranial sutures that leads to changes in the skull shape and the appearance of the facial skeleton. Dysmorphic features are not the only symptom of genetic diseases. Other symptoms associated with genetic disorders, such as intellectual impairment, autism, and organ anomalies have a crucial role in defining the severity of the disease, the diagnostic scheme, and disease management. Dysmorphic assessment is an essential part of a genetic consultation. However, it requires much experience and is often very individual.

Recently, high-throughput molecular biology techniques, such as NGS, have been introduced into clinical testing. This extended the number of indications for genetic consultations and genetic trials. However, in many countries, the number of trained clinical geneticists is insufficient, e.g., in Poland, a country with a population of 38 million, there are 146 clinical geneticists. In some national genetic centers, the waiting time for an appointment is 2.5–3 years. Moreover, the diagnostic yield of genetic testing is still limited in many ailments. For example, congenital heart disease is the most common birth defect, affecting nearly 10 to 12 per 1000 live-born infants (1–1.2%). The diagnostic yield of high-throughput genetic testing in nonsyndromic congenital heart disease is only ~10%. In syndromic cases, where heart disease is associated with other abnormalities, the success rate of genetic testing increases by ~30% [4,5]. Therefore, it seems crucial that the patients that are referred to clinical genetic units are selected correctly.

We performed a series of experiments to evaluate whether computer-aided facial analysis techniques could be used to detect genetic syndromes with dysmorphic features. Many studies that have been performed in the field were focused on the challenging task of establishing diagnosis based on facial pictures only. As there are still new genetic diseases to be discovered, in the approach we undertook, we have focused on the ability of the system to classify as abnormal syndromes that were not present in the trial dataset. The multiclass classification into 15 diseases and a control class had the best accuracy of 84% in our setup. The binary classification (disease vs. control) accuracy of ~96% was achieved on our dataset composed of patients suffering from 15 genetic diseases and control individuals. The system performed well in classifying patients suffering from diseases that were not removed from the training dataset, indicating that a sensitive screening tool is actually very feasible.

The system presented can be easily moved to the Raspberry Pi computer platform. Various components, such as a high-quality camera, can be attached to the Raspberry Pi. The camera would take a photo of the patient, and then a neural network would analyze the features of the picture to classify for genetic disease. Such a solution will allow the implementation of a system that operates on a mobile device, which we are currently working on.

## 2. State of the Art

The diagnostic analysis of facial pictures started with manual annotation of landmark points and use of neural networks for binary classification of images into Down syndrome and healthy controls [6]. A geometric analysis of 2D landmark points with local texture information enabled classification of patients with six disorders with a relatively high accuracy of 75% [7]. However, subsequent analysis performed by the same authors revealed that the classification success depends on the condition in which the picture was taken [8]. Balliu et al. analyzed 205 images of patients with 14 diseases [9]. Manually localized landmark points were processed by Delaunay triangulation. The distances and angles of the resulting net were used to perform classification with multinomial regression and an elastic net penalty with an accuracy of 62%. Automatic landmark detection methods such as Constrained Local Model and Active Appearance Model were also successfully applied in the genetic field [10]. For example, Zhao et al. achieved 96% binary classification accuracy for Down syndrome, and 97% for 24 syndromic subjects and 80 healthy subjects [11]. Support vector machines (SVM) [12] trained with 44 automatically detected landmark points recognized Noonan syndrome with 89% accuracy [13]. A system trained with 130 points, and texture information encoded using local binary patterns, outperformed a trained clinician in classifying patients with Cornelia de Lange (87% vs. 77%) [14]. In a seminal paper, Ferry et al. applied an Active Appearance Model for average syndrome face feature extraction with clustering, to classify patients with eight syndromes with very high accuracy [15]. Moreover, the authors showed that patients with diseases unknown to the system might be closer to the affected than to the controls in the so-called Clinical Face Phenotype Space. DeepGestalt is an algorithm-based convolutional neural network that uses 26,190 pictures of patients [16]. DeepGestalt achieves a 91%, top-10 accuracy in identifying over 215 genetic syndromes and is implemented in Face2Gene software, that is currently the most widely used application in clinical settings. 

## 3. Materials and Methods

### 3.1. Picture Database

Facial images of patients suffering from 15 genetic disorders were collected by searching PubMed and social media sites of foundations and organizations devoted to specific diseases. The resulting dataset was composed of 101 pictures of patients with 22q11 microdeletion syndrome, 90 with Angelman syndrome, 25 with Coffin–Lowry syndrome, 35 with Cornelia de Lange syndrome, 33 with Crouzon syndrome, 86 with Down syndrome, 26 with Fragile X syndrome, 118 with KBG syndrome, 31 with Kabuki syndrome, 63 with Mowat–Wilson syndrome, 86 with Noonan syndrome, 65 with Pitt–Hopkins, 112 with Smith–Lemli–Opitz syndrome, 38 with Wideman–Steinert syndrome and 32 with Williams syndrome. Face images of individuals aged between 5 and 12 years old from the UTKFace were used as the control dataset [17]. In addition, for binary classification into patients and healthy controls, 2101 pictures were available, with 941 individuals suffering from one of the genetic diseases and 1160 healthy individuals.

### 3.2. Face Classification

**Figure 1:**
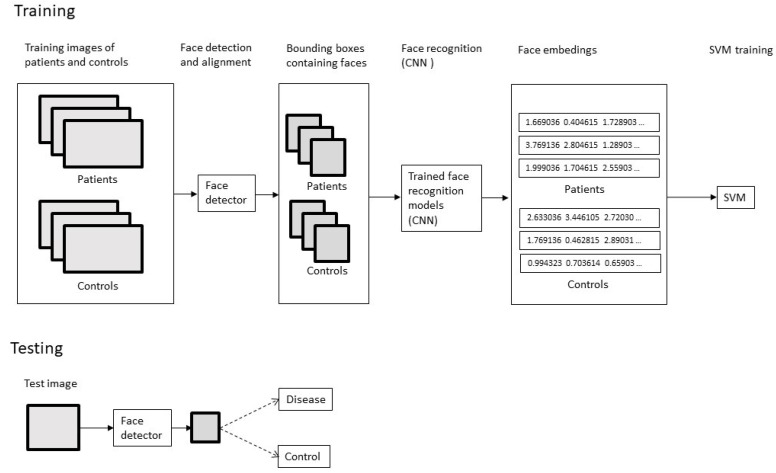
Diagram of the system architecture.

In the first stage of face classification (Figure 1), a detector was used to localize the coordinates of a box containing the face and align it. Face detection and alignment is a computer vision problem that involves finding faces in photos and obtaining a canonical alignment of the face based on geometric transformation. We evaluated two types of detectors: Multi-task Cascaded Convolutional Neural Network (MTCNN) [18] and a detector based on histograms of oriented gradient (HOG) [19] implemented in the dlib library [20]. Briefly, the method based on HOG divides an image into small connected cells, computes a histogram for each cell, and combines the histograms into a unique feature vector. In the last stage, the support vector machine’s learning algorithm is trained with feature vectors to perform face detection in test images. The HOG-based detector is known to perform well on frontal images. However, since our dataset also contained images with some degree of head rotation, we evaluated the performance of one of the deep learning methods introduced more recently, and have achieved very good results on benchmark face detection datasets. We used MTCNN, a state-of-the-art algorithm that has been shown to perform well on images taken in an unconstrained environment, with various poses, illuminations, and occlusions. MTCNN uses three convolutional neural networks: P-Net, R-Net, and O-Net. In the first step, an image is resized to create multiple copies of different sizes. The copies are fed into the P-Net, that generates candidate bounding boxes containing faces. Low confidence boxes are rejected. Next, the number of boxes is reduced by the Non-maximum suppression (NMS) method, based on the confidence score and position in the original image coordinate system. The process of NMS is based on bounding boxes, and merging regression in the second stage, involving the R-Net. In the last step, involving the O-Net, high-confidence face bounding boxes and 5 landmark points are generated. Next, several types of neural networks trained in face recognition were used to create face embeddings. Deep Learning methods, especially Convolutional Neural Networks (CNNs), have been shown to be very successful in many computer vision problems such as recognition and classification of images. Multilayer neural networks are able to learn spatial hierarchies of features automatically through the backpropagation algorithm. Convolutional Neural Networks outperform other face recognition methods on many benchmark datasets. They generate a face embedding feature vector that describes the analyzed face, and can be directly used for comparison and identification. We utilized the DeepFace framework, and evaluated the performance of 7 state-of-the-art deep learning models: VGG-Face, Facenet, Facenet512, OpenFace, DeepFace, DeepID, and ArcFace [21]. Face embedding is a vector that represents the features extracted from the face, and can be used for face recognition, face identification, and clustering to group like-faces. Generated face embeddings were used to train classifiers in two scenarios: a binary problem consisting in classification into disease group and normal group, and a multiclass problem responsible for detecting a specific genetic syndrome. A support vector machine (SVM) classifier was used for both tasks. Support vector machines are a set of supervised learning algorithms that can be used for classification, regression, and outliers detection. SVMs are based on construction of a hyperplane that best divides a dataset into classes. One of the main advantages of the SVMs is that they are effective in cases where number of dimensions is greater than the number of samples. The dataset was split into training (70%) and test (30%) sets and fed into a SVM with a linear kernel. Standard metrics such as accuracy, precision, and recall were calculated to evaluate the performance of the models. Due to the study’s exploratory nature, the classification was repeated five times, and average values were reported. In the case of the binary problem, to assess whether the system was able to classify “unknown diseases” correctly, each of the syndromes was removed from the training set. Next, the classification testing was performed only on the patients with the specific syndrome that was never presented to the classifier.

### 3.3. Geometric Analyses

The 3D Face Alignment Network detected 68 3D landmark points from 2D images [22]. The algorithm is based on a neural network that was trained on a large dataset of 2D face images and annotated 3D coordinates of landmark points. For each analyzed 2D image, the network output consisted of 68 coordinates in three dimensions aligned in a unified coordinate system. A total of 16 landmark points and 11 distances between them were selected to represent the main face areas analyzed during dysmorphology assessment (Figure 1). For selected point pairs in 3D space, the length of a line segment between the two points was calculated for every image in the dataset. The distances are expressed in the units of the coordinate system. In the case of symmetric distances, an average was calculated. Finally, the distances were used to train a support vector machine-based classifier in the two scenarios described in the face classification section.

## 4. Results

**Figure 2:**
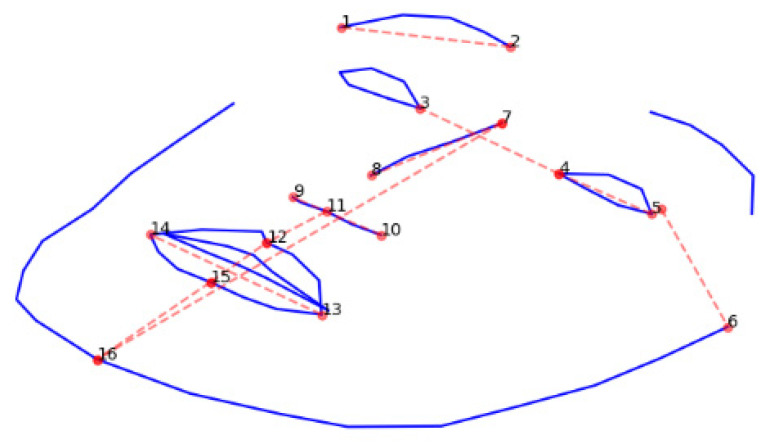
Localization of landmark points that were used for automated anthropometric measurements.

The results of automatic anthropometric measurements based on localization of 3D landmark points on the face (Figure 2) can be found in Table 1. The points were selected based on low standard deviation, and these reflected the main areas of the face analyzed during physical examination. Automated anthropometric measurements detected several characteristic features of genetic syndromes. For example, hypertelorism, an abnormally large distance between the eyes, was reflected by the increased distance between the corners of the eyes in Crouzon syndrome, Mowat–Wilson syndrome, Noonan syndrome, or Wideman–Steinert syndrome. In contrast, in Fragile X syndrome, this distance was smaller than in the controls, indicating hypotelorism, according to clinical genetic textbooks. Similarly, a short nose was present in Cornelia de Lange and Down syndrome. However, the performance of a support vector machine trained with these measurements was unsatisfactory (multiclass task accuracy 56%, binary task classification accuracy 67%).

The results of the multiclass classification system based on deep learning face recognition models are shown in Table 2. From the seven models tested on the dataset, four achieved an accuracy of more than 70%. The best result of 84% accuracy was obtained with a classification system based on the MTCNN detector and SVM trained with embeddings generated by the ArcFace model. 

**Figure 3:**
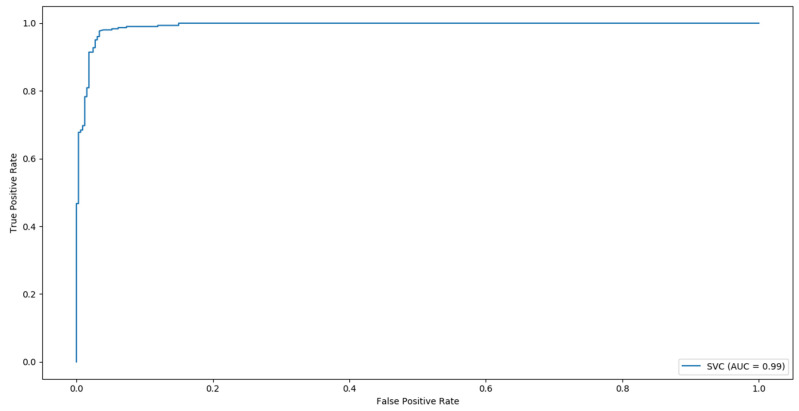
Receiver operating characteristic curve for the two-class classifier based on the DeepFace model.

In binary task classification into disease and control groups, SVM based on MTCNN detector and DeepFace embeddings was the most successful, with an accuracy of 96% (Table 3). In addition, the classifier performed well in classifying as abnormal patients with diseases that were not presented during training (Table 4, Figure 3).

## 5. Discussion

We have evaluated several approaches to construct a classifying system for detecting genetic disease from images of the face. There were significant differences between models generating face embeddings in our experiments. We achieved the best results in multiclass problems with ArcFace. DeepFace gave the best results for the two-class classification. The best accuracy of a multiclass classifier detecting one of 15 genetic syndromes was 84%. The two-class classifier, intended to detect the presence or absence of genetic disease, had an accuracy of 96% in our experiments. More importantly, the binary classifier detected disease features in patients with diseases that were not present in the training dataset. Thus, the classifier was able to generalize differences between patients and controls, and detect abnormalities without the need for information about the specific disorder. This indicates that the system does not have to be trained with all genetic diseases to detect ‘genetic’ features of a face and, therefore, may be used as a first-line screening tool. 

A machine learning face recognition system outperformed a simple approach based on our experiments’ geometric characteristics of face landmark points. The clinical examination performed by a trained dysmorphologist is based on individual experience [2]. A trained eye detects single distances between points on the face and relations between them. Moreover, automated measurements from pictures taken in uncontrolled conditions require normalization by one of the measurements, affecting the natural geometry of the face. The images used in the study were collected from many sources, with a variable degree of head rotation and various facial expressions, which could affect the landmark detection and measurements [8]. Machine learning-based face recognition models were trained using millions of unstandardized images. They are applied in face identification that requires extraction of many interrelated features, which resembles dysmorphic evaluation. Therefore, these models are more suitable as computer aided clinical face analysis tools. 

Face2Gene is a state-of-the-art tool that clinicians use in many countries. The solution was built based on a database of 26,000, including patients with more than 200 syndromes. The application was designed to point out the most probable diseases, with a score labeled as high, medium, or low [16]. However, the evaluation requires manual submission of pictures; therefore, it is difficult to use in a comparative way. In addition, Face2Gene attempts to solve a more challenging task due to the number of symptoms. We have submitted to Face2Gene a random sample of 200 control pictures from our dataset, and 69% had no diagnostic high or medium suggestions. However, 31% of control subjects would be erroneously assigned to the disease groups. This indicates that Face2Gene is designed for a different task and, without modification, might not perform well as a screening tool. 

A single dysmorphic feature is usually not unique for a specific disorder. For example, hypertelorism is not only present in Coffin–Lowry syndrome but also, among others, in Crouzon syndrome. This overlap in dysmorphic features most probably enables the correct classification of patients with a disease unknown to the system in the binary task. The system analyzes only face image information without knowledge about symptoms, other test results, or family history. Seventy-two percent of rare diseases have a genetic background, while others result from infections, allergies, and environmental causes. One of the limitations of our study is the lack of training on images of patients with non-genetic etiologies. There are usually no dysmorphic features in these cases, and therefore, facial images are not published and available. Fetal alcohol syndrome (FAS), caused by an environmental factor, is associated with specific dysmorphic changes, and represents an example of a disease that could be misclassified as a genetic syndrome. Therefore, cases of such diseases should be included in the final training dataset before clinical application. However, it is essential to note that in the case of a screening system, the number of patients with genetic diseases classified as control is more concerning than a false positive misclassification.

Ethnic background and age are essential factors in face recognition and genetic syndrome classification. Ethnicity has been shown to have a high impact on the performance of Face2Gene in the classification of Down syndrome patients of Caucasian and African origin [23]. There is a bias in patients’ ethnicity described in the literature, and, therefore, the availability of face images. The screening system described in this paper should be constructed using images reflecting the local population and its ethnic structure. In many clinical genetic units, face pictures are routinely taken and could be used as a data source. Information concerning the exact age at the picture taking could improve the performance of dysmorphic features analysis. However, for many pictures from the literature, information about the age was not available. We, therefore, limited the analyses to pictures arbitrarily labeled as children or young adolescents. 

For many countries, the time needed for diagnosis is counted in years, particularly for many genetic diseases. This is due to the complex nature of genetic disorders, the high cost of laboratory testing, and limited human resources, i.e., trained clinical geneticists. Moreover, most of the patients that have genetic tests remain undiagnosed despite significant progress in the field. For example, a majority of patients with isolated congenital heart disease will have normal results in genetic tests. However, if the heart disease is not isolated and there are additional symptoms or dysmorphic features, the probability of a positive test is significantly increased by ~30% [4,5,24]. The so-called ‘diagnostic odyssey’ of a patient with suspicion of a genetic disease usually starts from an appointment with a general practitioner or pediatrician. Then, the doctor has to decide whether to refer the patient to a clinical genetic unit. In many countries, the resources in the field of clinical genetics are minimal. A sensitive screening system would enable the selection of patients for genetic referral and reduce the patient’s time to wait for the diagnosis. The number of patients with a genetic syndrome classified as controls was ~4% in the analysis based on the DeepFace model. For the misclassified patient, that would mean a potential delay in diagnosis. The interpretation of this percentage depends on the capacity of clinical genetic units in a given country.

Our analyses were based on a relatively small dataset of 15 disorders out of thousands of genetic syndromes. However, the same approach with an extended dataset could have clinical application. Furthermore, once the training of the deep learning algorithm is performed, the testing of individual images is fast, and can be performed on a desktop computer.

It is important to remember that there are more than 7500 genetic diseases, and probably still hundreds to be discovered. Therefore, the screening system has to detect abnormalities associated with diseases that were unknown at the time of its construction. Our results suggest that such a system is possible. However, a more extensive study, including a larger dataset with more diseases, is needed.

## Figures and Tables

**Table 1:** Automated 3D anthropometric measurements taken from 2D images in a control group and 15 genetic syndromes. The average distances between points that are labeled in Figure 1 are given.

Patient Group	Distance between Points (Labeled in Figure 2)										
	1–2	3–4	4–5	5–6	7–8	9–10	11–12	13–14	12–15	15–16	7–16
Control	32.31	27.66	17.57	17.57	26.14	17.46	8.94	39.2	14.27	23.99	75.71
22q11 microdeletion syndrome	32.4	27.97	17.47	17.47	26.33	16.91	9	36.12	12.2	25.34	74.71
Angelman syndrome	31.8	26.76	16.88	16.88	25.2	17.72	8.59	39.27	18	23.62	76.69
Coffin–Lowry syndrome	33.17	30.37	17.61	17.61	26.01	17.89	9.78	38.93	24.01	22.41	83.27
Cornelia de Lange syndrome	32.21	28.52	17.25	17.25	23.49	16.6	10.73	36.3	11.73	22.93	71.67
Crouzon syndrome	33.52	30.33	18.4	18.4	25.45	16.92	8.26	35.9	15.96	24.29	75.28
Down syndrome	31.93	27.64	17.11	17.11	24.04	17.19	8.78	37.84	14.74	22.35	71.78
Fragile X syndrome	32.64	27.45	17.8	17.8	27.44	17.69	9.55	39.94	19.26	23.67	82.06
KBG syndrome	32.3	28.31	17.46	17.46	25.14	16.69	10.31	36.23	13.98	24.68	76.23
Kabuki syndrome	32.83	28.76	17.82	17.82	25.39	16.77	10.09	34.46	13.67	24.03	74.45
Mowat–Wilson syndrome	32.74	28.53	17.72	17.72	27.16	17.76	8.82	39.74	16.18	24.91	79.38
Noonan syndrome	32.87	28.71	17.65	17.65	25.71	16.9	10.25	35.34	14.46	24.47	76.41
Pitt–Hopkins syndrome	32.34	27.67	17.35	17.35	25.93	17.76	8.34	38.77	17.47	23.44	76.67
Smith–Lemli–Opitz syndrome	32.24	27.89	17.16	17.16	24.38	17.38	9.43	38.09	20.56	22.47	78.14
Wideman–Steinert syndrome	32.72	29.07	17.15	17.15	26.45	16.96	9.4	36.89	15.95	23.69	77.52
Williams syndrome	31.98	27.77	17.52	17.52	24.52	17.28	10.72	39.3	16.69	22.12	76.07

**Table 2:** Result of multiclass classification for 4 most accurate face recognition models.

Patient Group	Face Recognition Model							
	ArcFace		FaceNet		DeepFace		FaceNet512	
	Precision	Recall	Precision	Recall	Precision	Recall	Precision	Recall
22q11 Microdeletion syndrome	0.690	0.755	0.545	0.588	0.484	0.580	0.493	0.517
Angelman syndrome	0.657	0.680	0.396	0.576	0.467	0.669	0.419	0.525
Coffin–Lowry syndrome	0.811	0.634	0.742	0.663	0.417	0.343	0.537	0.761
Control	0.910	0.956	0.890	0.898	0.944	0.979	0.859	0.883
Cornelia de Lange syndrome	0.949	0.811	0.593	0.649	0.701	0.394	0.856	0.750
Crouzon syndrome	0.907	0.838	0.783	0.915	0.748	0.459	0.871	0.866
Down syndrome	0.911	0.819	0.755	0.756	0.537	0.632	0.770	0.699
Fragile X syndrome	0.534	0.313	0.397	0.513	0.436	0.369	0.203	0.237
KBG syndrome	0.786	0.714	0.669	0.610	0.599	0.708	0.580	0.560
Kabuki syndrome	0.787	0.631	0.564	0.474	0.495	0.391	0.789	0.576
Mowat–Wilson syndrome	0.902	0.839	0.777	0.665	0.501	0.326	0.777	0.793
Noonan syndrome	0.562	0.543	0.529	0.453	0.362	0.353	0.558	0.494
Pitt–Hopkins syndrome	0.783	0.610	0.568	0.373	0.284	0.225	0.544	0.383
Smith–Lemli–Opitz syndrome	0.683	0.711	0.562	0.572	0.599	0.517	0.650	0.619
Wideman–Steinert syndrome	0.750	0.813	0.774	0.661	0.601	0.483	0.788	0.586
Williams syndrome	0.889	0.728	0.694	0.542	0.533	0.218	0.692	0.591
Accuracy		0.846		0.762		0.757		0.746

**Table 3:** Result of two-class classification for the 4 most accurate models.

Patient Group	Face Recognition Model							
	DeepFace		ArcFace		DeepID		FaceNet	
	Precision	Recall	Precision	Recall	Precision	Recall	Precision	Recall
Control	0.961	0.971	0.922	0.922	0.940	0.921	0.894	0.915
Disease	0.962	0.949	0.907	0.908	0.900	0.922	0.894	0.867
Accuracy	0.961		0.915		0.922		0.894	
False positives	0.04		0.05		0.08		0.07	
False negatives	0.04		0.09		0.09		0.1	
Negative redictive value	0.957		0.915		0.914		0.906	

**Table 4:** Two class classification results for test sets of patients with diseases that were removed from the training data.

Disease Removed from Training	DeepFace Using 70% of the Dataset without Given Disease for Training	DeepFace Using 100% of the Dataset without Given Disease for Training
22q11 microdeletion syndrome	0.70	0.88
Angelman syndrome	0.85	0.94
KBG syndrome	0.84	0.98
Down syndrome	0.92	0.97
Crouzon syndrome	0.91	0.88
Cornelia de Lange syndrome	0.85	0.91
Noonan syndrome	0.83	0.99
Williams syndrome	0.91	1.00
Fragile X syndrome	0.65	1.00
Kabuki syndrome	0.90	0.94
Mowat-Wilson syndrome	0.92	0.94
Coffin–Lowry syndrome	0.96	1.00
Smith–Lemli–Opitz syndrome	0.79	0.95
Pitt–Hopkins syndrome	0.79	0.91
Wideman–Steinert syndrome	0.84	0.97
